# New insights into anti-Müllerian hormone role in the hypothalamic–pituitary–gonadal axis and neuroendocrine development

**DOI:** 10.1007/s00018-020-03576-x

**Published:** 2020-06-20

**Authors:** Mauro S. B. Silva, Paolo Giacobini

**Affiliations:** Univ. Lille, Inserm, CHU Lille, U1172-LilNCog-Lille Neuroscience and Cognition, 59000 Lille, France

**Keywords:** AMH, Fertility, GnRH, PCOS, CHH

## Abstract

Research into the physiological actions of anti-Müllerian hormone (AMH) has rapidly expanded from its classical role in male sexual differentiation to the regulation of ovarian function, routine clinical use in reproductive health and potential use as a biomarker in the diagnosis of polycystic ovary syndrome (PCOS). During the past 10 years, the notion that AMH could act exclusively at gonadal levels has undergone another paradigm shift as several exciting studies reported unforeseen AMH actions throughout the Hypothalamic–Pituitary–Gonadal (HPG) axis. In this review, we will focus on these findings reporting novel AMH actions across the HPG axis and we will discuss their potential impact and significance to better understand human reproductive disorders characterized by either developmental alterations of neuroendocrine circuits regulating fertility and/or alterations of their function in adult life. Finally, we will summarize recent preclinical studies suggesting that elevated levels of AMH may potentially be a contributing factor to the central pathophysiology of PCOS and other reproductive diseases.

## Introduction

Gonadal sex differentiation is under the control of a primary sex determination program, governed by the genetic code, and of a secondary sex determination program, mainly dictated by hormones. The undifferentiated gonadal primordium is bipotential in mammals and sex-determination signals are required to drive the full presentation of male and female phenotypes. Anti-Müllerian hormone (AMH), formerly named Müllerian inhibiting substance (MIS), is a critical testicular signal driving the development of the male reproductive tract. In the late 1940s and early 1950s, Professor Alfred Jost pioneered the notion that sexual determination of the male genitalia was driven by AMH and testosterone that act in concert to suppress the development of the Müllerian ducts and virilize the progression of Wolffian ducts, respectively [1, 2]. The masculinizing effects of AMH were also classically known to drive Freemartinism, a severe form of virilization and infertility of mammalian females due to shared chorionic vascular anastomoses with male twins [3].

The notion that AMH had only male-specific functions has undergone a paradigm shift once AMH was found to be synthesized and secreted by the ovaries during postnatal life and be actively involved in ovarian follicular development [4]. Furthermore, a rising number of reports demonstrate that AMH actions reach beyond the gonadal function targeting brain circuits [5–7] and pituitary secretory molecular machinery [8, 9]. This postulates that AMH may have important neuroendocrine roles contributing to reproductive fitness by acting at different levels of the Hypothalamic–Pituitary–Gonadal (HPG) axis. The control of reproduction is highly dependent upon neural circuits in the brain. Gonadotropin-releasing hormone (GnRH) neurons, located in the hypothalamus, provide the final output from the brain to the pituitary gland, where GnRH stimulates the secretion of follicle-stimulating hormone (FSH) and luteinizing hormone (LH). These gonadotropins, in turn, operate downstream on the gonads to coordinate gonadal development and production of steroid hormones in both sexes, and ovulation in females. Sex steroids hormone feedback to GnRH neurons modulating the pace of their activity and GnRH secretion [10].

As GnRH neurons express AMH [11] and its receptors [6], along with the presence of AMH signaling in gonadotropes [12, 13] and gonads [14, 15], the present review will focus into recent findings of novel AMH actions along the HPG axis. This review also aims to convey classical pharmacological studies and more recent work employing transgenic mouse technology dissecting out cellular and molecular mechanisms revealing the role of AMH on reproductive function and in diseases affecting fertility.

## AMH signaling

AMH is a homodimeric glycoprotein with disulfide-linked subunits belonging to the Transforming Growth Factor β (TGF-β) superfamily. The human *AMH* gene encodes a pre-proAMH molecule bearing 560 amino acids and it is located in the chromosome 19p13.3 [16]. Proteolytic cleavage of the pre-proAMH produces proAMH homodimer, a 140-kDa biologically inactive precursor that can be found in situ, for instance in the ovarian follicular fluid [17], and in the general circulation [18]. Thereafter, proAMH undergoes further proteolytic cleavage by proteases yielding the biologically active form of AMH composed by a 25-kDa C-terminal and a 110-kDa N-terminal dimer, which are non-covalently bound [19], being commonly named AMH_N,C_. The C-terminal domain drives the biological activity of AMH, whereas the N-terminal domain seems to be responsible for potentiating the activity of AMH_N,C_ [20].

AMH specifically binds to the AMH type II receptor (AMHR2) with high affinity [21]. The human AMHR2 harbors 573 amino acids with an extracellular domain, which interacts with AMH, and an intracellular domain presenting weak autophosphorylation activity [22]. This activity is enhanced when AMHR2 heterodimerizes with different AMH type I receptors (AMHR1) that belong to the Bone Morphogenetic Protein Receptor (BMPR) class such as activin-like kinase 2 (ALK2) (or activin A receptor type 1; ACVR1), ALK3 (or BMPR1a) and ALK 6 (or BMPR1b) [23]. Among these receptors, ALK3 is required for the regression of Müllerian ducts in the male reproductive tract [24], whereas ALK6 is required for the full reproductive competence in both male and female mice [23, 25]. AMH binding to its receptors transduces intracellular signaling through Smad proteins, β-catenin pathway and nuclear factor κB (NFκB) pathway [23]. Following the binding of AMH, interactions between AMHR2 and AMHR1 lead to the activation of Smad proteins, which are translocated into the nucleus to promote the transcriptional and physiological effects of AMH [23]. More recent evidence shows that AMH induces changes in microRNA levels promoting downstream silencing of targeted genes in murine ovaries to regulate folliculogenesis and neuroendocrine modulation through FSH actions [26].

AMH is mainly secreted by the gonads in different vertebrates, from teleost fish [27] to avian [28] and mammalian species [17, 20]. AMH expression is sexually differentiated according the developmental period in mammals, which is likely to be associated with the time of gonadal maturation. Males produce abundant levels of AMH during embryonic life, which is synthetized by Sertoli cells of the testis, and this production declines over the pubertal period until a strong decrease of testicular AMH synthesis over adulthood [4]. This is due to the inhibitory effect of AMH on the development of the female reproductive tract in male embryos [1, 2]. During early postnatal life, Sertoli cells are finally organized into seminiferous tubules and spermatids promptly undergo meiosis, and these events are coincident with an abrupt decline of testicular AMH production [4]. Females express detectable levels of AMH following birth in ovarian granulosa cells of growing follicles, which increase during infantile period and are maintained at high levels following puberty until menopause, when AMH secretion drops because of the exhaustion of the growing follicle pool [4]. AMH secretion and action are critical for the folliculogenesis over reproductive life.

Although gonadal AMH expression declines in males during postnatal life, tissue-specific AMH synthesis may remain over adulthood in other tissues. It has been reported that adult motor neurons secrete and respond to AMH in an autocrine/paracrine manner in the central nervous system of mice [29]. In addition, adult neurons in the forebrain produce AMH and effectively respond to AMH actions changing their electrical behavior, such as increasing synaptic potentiation in hippocampal neurons [30]. Therefore, different levels of circulating AMH throughout development and adult life, as well as different levels of tissular expression of AMH and AMH receptors may define the impact of AMH signaling in reproductive and non-reproductive tissues.

## Role of AMH in the hypothalamus and pituitary

The control of reproduction in vertebrates is ultimately dependent upon neural circuits in the brain. Brain circuits within the hypothalamus in synergy dictate the proper functioning of GnRH neurons, which are the final output to the control of gonadotropin secretion. To achieve this, GnRH neurons release GnRH peptide in a pulsatile manner into the hypophyseal portal system. Thereupon, pulsatile GnRH signal promotes the secretion of FSH and LH from the pituitary gland into the systemic blood stream. In females, FSH and LH govern downstream actions that coordinate ovarian follicle development, ovulation and the production of ovarian steroid hormones, such as estrogens, progesterone and androgenic precursors. In males, the two gonadotropins will keep in check spermatogenesis and, mostly, the synthesis of testosterone by Leydig cells in the testis. Completing a feedback system, gonadal steroid hormones act upon steroid-sensitive hypothalamic circuits that pace GnRH neuron activity and GnRH secretion [31, 32]. These various steroid-sensitive neuronal circuits “sense” and transmit homeostatic cues to GnRH neurons and modulate a pulsatile GnRH secretion to ensure fertility [33].

### AMH signaling in GnRH neuronal development and function

GnRH neurons display peculiar characteristics from the time they are born to their final locus within the hypothalamus. The ontogenesis of GnRH neurons and the olfactory system are remarkably associated. GnRH neurons originate and differentiate in the medial part of the olfactory placode of the developing nose, namely the vomeronasal organ (VNO) [34], and migrate along the nervus terminalis and along the vomeronasal nerve into the developing forebrain [35, 36]. In humans, GnRH-expressing cells are firstly detected in the VNO and migration into the forebrain begins during the 6th post-conception week [37]. Hypophysiotropic GnRH neuron cell bodies reach their final location in a scattered distribution throughout the anterior portion of the hypothalamus, and these neurons extend long projections reaching the median eminence where GnRH peptide is secreted [38, 39].

**Fig. 1:**
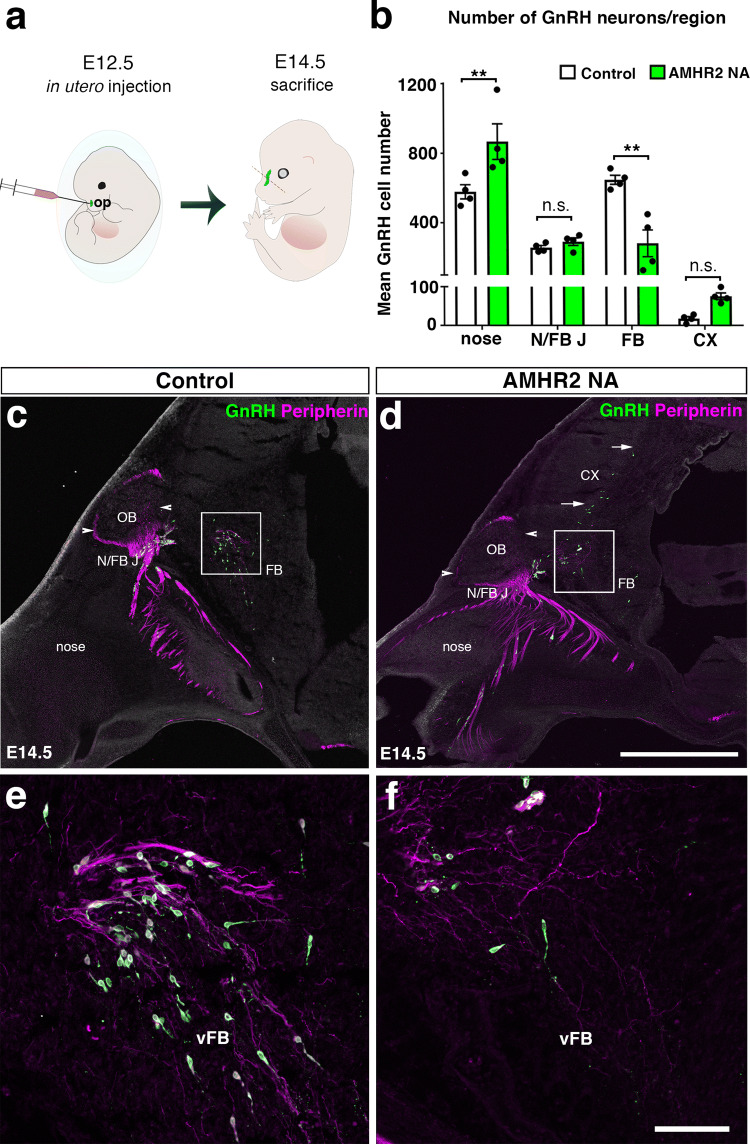
Neutralization of AMHR2 disrupts GnRH neuronal migration in mouse embryos. **a** Schematic of in utero injections of the neutralizing antibody anti-AMHR2 (AMHR2 NA) into the olfactory pits of mouse embryos. Injections were performed at E12.5 and embryos harvested 48 h later. **b** Quantitative analysis of GnRH neuronal distribution throughout the migratory pathway in the two experimental groups. Data are represented as mean ± s.e.m (*n* = 4, two-way ANOVA, *F*_3,24_ = 15.09, *P* < 0.0001; followed by Holm-Šídák multiple comparison post hoc test, **P < 0.005; n.s., not significant, P > 0.05). Data are represented as mean ± s.e.m (*n* = 4, unpaired two-tailed Student’s *t* test: mean cell number, *t*_6_ = 0.3796, *P* = 0.7173). **c**–**f** Representative photomicrographs of sagittal sections of mouse embryos injected at E12.5 with either saline or AMHR2 NA and immunostained for GnRH (green) and Peripherin (magenta) at E14.5. **e**,** f** Higher magnification confocal photomicrograph of boxed areas in **c** and **d**. *Cx* cortex, *FB* forebrain, *N/FBJ* nasal/forebrain junction, *oe* olfactory epithelium, *NMC* nasal mesenchyme. Scale bars: **c**, **d** 2.5 mm; **e**, **f** 50 μm. Adapted and reproduced with permission from Malone et al. [11]

Various chemosignals influence GnRH neuron migration such as cell adhesion molecules, neurotransmitters and growth factors. Recent studies show that GnRH neurons themselves produce AMH from embryonic life (E12.5) in mice, and that AMH expression in these neurons display a steady increase over time toward adulthood [11]. Human fetal GnRH neurons also express AMH throughout their migratory route from the nose to the developing forebrain [11]. AMHR2 is also expressed in migratory GnRH neurons, in mice and human foetuses [6]. Pharmacological blockade of AMHR2 using in utero injection of AMHR2-neutralizing antibody into the E12.5 mouse olfactory pit results in the arrest of GnRH neurons in the nose and in the disruption of their migratory process [11]. Interestingly, this pharmacological blockade affects olfactory/vomeronasal nerve targeting to the hypothalamus, which is likely to contribute to the abnormal GnRH neuron migration (Fig. 1). AMHR2 homozygous knockout mice display a decreased number of GnRH neurons in the rostral preoptic area (rPOA), which is associated with lower LH secretion in females and impaired reproductive function in both sexes [11]. When evaluating which AMHR1 co-participates in these AMH actions, in vitro studies demonstrated that BMPR1b (a.k.a. ALK6) is expressed and required for normal cell motility in migratory GnRH neurons [11]. Together, these observations determined that AMH signaling is required to properly guide GnRH neurons throughout their migratory pathway until their final target areas in the hypothalamus.

**Fig. 2:**
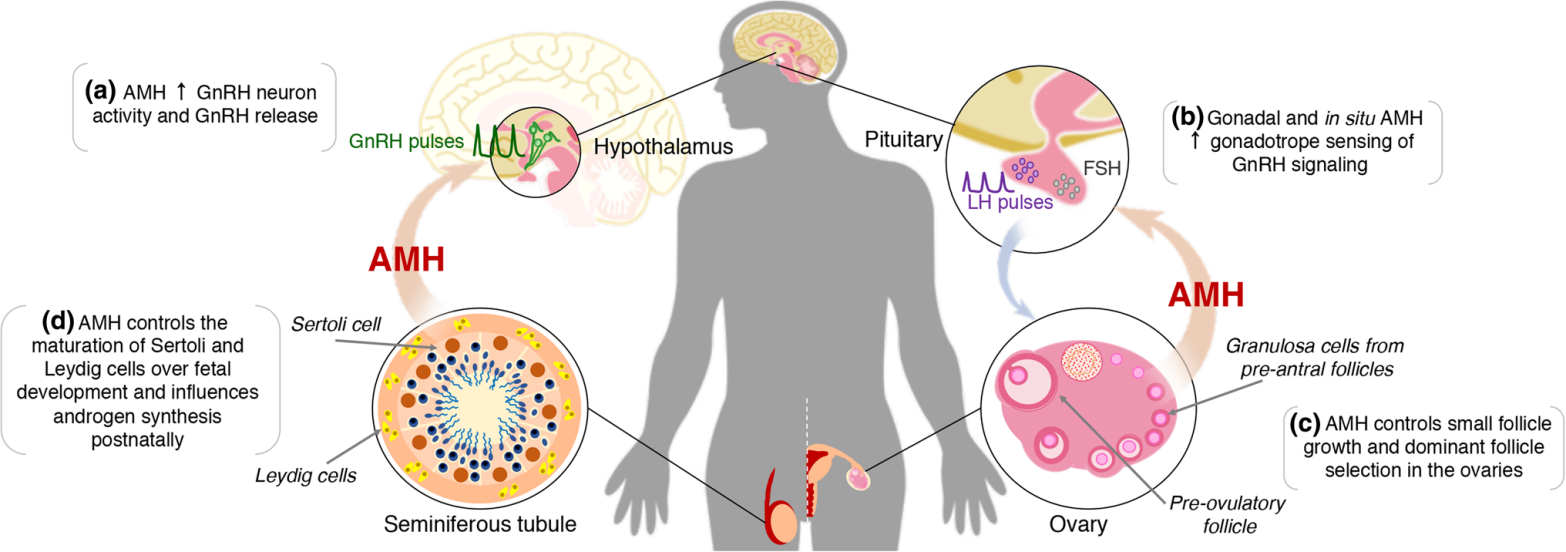
AMH actions in the hypothalamic–pituitary–gonadal (HPG) axis. Novel evidence shows that AMH can act centrally increasing the activity of GnRH neurons (**a**), which ultimately modulate GnRH/LH secretion. In the pituitary (**b**), AMH enhances FSH synthesis and elevates LH production following GnRH priming in the gland. AMH is mainly produced by the granulosa cells of small ovarian follicles in females (**c**). AMH actions within the ovaries control the small follicle growth and the selection of the dominant follicle. In males, AMH is mainly produced by Sertoli cells of the testes over embryonic and early postnatal period (**d**). Through paracrine actions, AMH drives the differentiation of the male internal genitalia and spermatogenesis. AMH also acts in Leydig cells controlling LH-mediated androgen production. Gonadal AMH levels may reach the brain and the pituitary to exert its endocrine actions; however, tissular AMH and AMH receptor expression indicate that autocrine regulations occur upstream in the HPG axis

The control of adult GnRH neuron excitability is defined through a fine tune regulation of extrinsic and intrinsic properties that will ultimately drive episodic GnRH secretion. The major recent advances in the field of neuroendocrinology of reproduction point to a great importance of hormone-sensitive afferent neurons, which control GnRH neuron excitation within the so-called GnRH neuron network [40–42]. Oppositely, fewer in vivo studies have elucidated direct hormonal actions on GnRH neurons influencing GnRH/LH secretion. As adult GnRH neurons express AMHR2, our group recently showed that AMH robustly increases GnRH neuron activity [6]. Electrophysiological recordings in vitro showed that AMH evokes a direct activation of GnRH neurons, which is dependent upon hormone concentration but independent of either sex or estrous cyclicity in female mice [6]. Further electrophysiological studies indicate that around 50% of GnRH neurons located in the rostral preoptic area (rPOA) respond with excitation to AMH [6] suggesting that there is a degree of heterogeneity within the GnRH neuron population regarding the influence of AMH actions in both sexes. As expected, peripheral AMH levels can activate GnRH neurons in vivo as demonstrated by the exogenous administration of AMH_N,C_, which induces c-fos expression, a surrogate marker of neuronal activation, in GnRH neurons in female mice [43] (Fig. 2a). Although AMHR2 is highly expressed in regions that are known to be important for the regulation of GnRH neurons such as the rPOA and the *organum vasculosum laminae terminalis* (OVLT), the exact nature of the cerebral cellular source remains to be determined. It is also unclear whether AMH can act in neurons that control the GnRH pulse generator, currently known to be arcuate nucleus (ARN) kisspeptin neurons [44, 45], and other neuronal afferents that control GnRH/LH secretion such as ARN GABAergic neurons [46] and ARN Agouti-related peptide (AgRP) neurons [47, 48]. In fact, intracerebral injection of AMH into the third ventricle of the brain, increases LH pulsatile secretion [6], suggesting that AMH may act upstream in different components of the GnRH neuronal network and modulate the GnRH pulse generation.

AMHR2 is also expressed by tanycytes [6], a glial cell type involved in the control of GnRH secretion into the hypophyseal portal system and which receives direct information about fluctuating levels of gonadal steroid hormones [49, 50]. Tanycytes regulate the access of GnRH neuron terminals to the fenestrated vessels of the median eminence (ME) through the ensheathment of GnRH neuron terminals to control the physical interaction between terminals and vessels dictating neuroendocrine output [49]. It has been shown that intravenously administered bioactive AMH can access the ME through the fenestrated vessels [43]. It is thus possible that peripheral AMH could act upon ME tanycytes, even though further studies are required to address this issue. To this point, novel evidence suggests that AMH may control GnRH secretion through direct influence on their neuronal electrical activity and/or through interactions with ME tanycytes, yet to be explored.

The relative contribution of locally produced and gonadal-derived AMH modulating GnRH output remains to be investigated. This warrants future investigations and could only be addressed generating novel conditional knock-out mice (brain-deficient and/or gonadal-deficient AMH transgenic animals), which are currently unavailable. In line with this notion, some recent findings started to shed light on the possible role of AMH locally produced in the brain in the modulation of neuronal activity, i.e. in the hippocampus [30]. Since AMH levels were reported to be higher in the plasma of adult female mice but not in the cerebrospinal fluid (CSF) as compared to males [30], it is possible to speculate that certain neuronal populations, like hippocampal neurons or GnRH neurons, produce AMH which, if secreted, could enter the CSF and potentially act as a paracrine factor via the CSF circulation in regulating functions of other brain structures, including the hypothalamus, which is known to express AMHR2 [6].

### AMH and the control of gonadotropin secretion

GnRH signaling in the pituitary is essential for the normal secretion of both gonadotropins, LH and FSH. The discovery of pulsatile nature of GnRH secretion in non-human primates and sheep revealed that GnRH and LH release are paired at a 1:1 pulse ratio [51] and that continuous GnRH administration inhibits gonadotropin secretion [52]. The pituitary is also responsible to regulate the amplitude, *i.e.* the magnitude, of LH pulses as it is determined by the frequency of GnRH stimulation from the brain and by the LH releasable secretory pool in the gland [53]. Although FSH synthesis is dependent upon GnRH signaling in the pituitary gland, this gonadotropin does not present a pulsatile secretion, which also seems to be independent upon GnRH pulses [54]. FSH and LH are closely related glycoproteins sharing and α-subunit and a unique β-subunit, both being under influence of GnRH signaling in the pituitary [55]. The synthesis of either gonadotropins is regulated by a synergistic relation between GnRH input and endocrine/paracrine factors such as activins, inhibins, among other TGF-β protein superfamily members [56].

Early evidence demonstrated that *Amhr2*^*−/ −*^ female mice present low levels of FSH during early adulthood [57], and oppositely, transgenic mice overexpressing human AMH (hAMH) exhibit high FSH and LH levels compared with wild-type mice [58]. Thereafter, Bédécarrats and colleagues showed that mouse gonadotrope-derived LβT2 cells express AMHR2 and that AMH promotes a steady elevation of FSH-β mRNA levels, whereas it does not affect LH-β mRNA synthesis [59]. Interestingly, in the presence of GnRH agonist, both FSH-β and LH-β promoter activity are enhanced by AMH [59], indicating that a priming effect of GnRH signaling dictates the effects of AMH on gonadotropin synthesis. The same research group also showed that AMH stimulates LH-β promoter activity in a dose-dependent manner when in the presence of GnRH agonist [59]. Both AMH and proAMH promote the activation of Smad1/5/8 pathway in LβT2 cells; however, proAMH shows a less potent effect when inducing Smad pathway activation and significantly increases FSH-β mRNA levels [8]. AMH is locally produced in the pituitary and *Amh* transcript levels are similar between female and male rats from postnatal period to adulthood [8]. Interestingly, the levels of *Amhr2* transcripts are sexually dimorphic throughout development in the rat pituitary. Female rats seem to synthetize more pituitary AMHR2 during prepubertal life, twice as much than males; *Amhr2* mRNA synthesis falls abruptly over adult life reaching lower levels than male counterparts [8]. Further in vivo work revealed that prepubertal female rats acutely treated with AMH substantially increase FSH levels, whereas prepubertal males do not respond to AMH [60]. Coupling this sexually dimorphic synthesis of AMHR2 and different sensitivity to AMH may dictate the response of the HPG axis before puberty. During infantile period, the HPG axis displays a transitory activation period called minipuberty, which is known to occur in humans, rodents and other mammals [61]. During this period, there is a substantial elevation of gonadotropin and gonadal steroid secretion similar to adult levels but without secondary sexual maturation [61]. In humans, this process is longer in girls (~ 2 years) than in boys, being associated with the first wave of ovarian follicle growth. In boys, minipuberty is shorter (~ 4–6 months) and linked to the postnatal maturation of the testes [61]. Therefore, it is likely that systemic and tissue-specific production of AMH may sustain the sex difference of gonadotropin secretion to properly prime the HPG axis for further maturation over pubertal development (Fig. 2b).

Gonadotropes are able to detect GnRH pulsatile release through a molecular machinery triggered by GnRH receptor (GnRH-R) signaling. The frequency by which GnRH-R signaling is stimulated by GnRH pulses from the hypothalamus defines a preferential release of either FSH or LH [55]. AMH does not increase *Gnrh-r* transcript levels [8]; however, new evidence shows that the frequency of GnRH-R signaling activation regulates *Amhr2* synthesis in the pituitary [9]. Using a cell culture perifusion system, researchers found that continuous GnRH exposure decreases *Amhr2* transcript levels, whereas low GnRH pulse frequency, known to stimulate FSH secretion, is ineffective in inducing such response [9]. In contrast, high GnRH pulse frequency delivery, known to favor LH secretion, promotes an increase of *Amhr2* expression, which presents a similar profile of high LH-β mRNA levels [9]. Further investigation revealed that GnRH enhances AMHR2 promoter activity through the activation of immediate early gene early growth response (Egr1), complementary to early reports showing that Egr1 participates in the upregulation of LH-β expression [62]. They also discovered that FOXO1 is required to decrease basal and GnRH-dependent AMHR2 promoter activity [9], which corroborates studies demonstrating that FOXO1 pathway downregulates FSH-β expression in female rodents [63]. Collectively, this indicates that GnRH actions in the pituitary regulate AMHR2 expression, which ultimately may influence a differential expression and secretion of LH and FSH by the pituitary.

## AMH and the regulation of gonadal function

It is without surprise that AMH actions in the gonads have been extensively studied and far more as compared to the other two upstream sites of the HPG axis. AMH is well known by its nourishing actions within the gonads sustaining the development of ovarian follicles in females and germ cells of the testes in males. The central role of AMH in gonadal function has grown from its initial sexual differentiation effects to how AMH coordinates gonadotropin and steroid hormone signaling, and beyond. We provide here a brief overview of the gonadal roles of AMH since these roles have been already thoroughly reviewed elsewhere (see reviews [64–67]).

### AMH is a gatekeeper of ovarian follicular development

AMH is mainly produced by the granulosa cells of small ovarian follicles, after the first recruitment of primordial follicles to its highest levels detected in pre-antral follicles [67]. The expression of AMH in granulosa cells of the follicle wall decreases during the transition to small antral follicles being absent for most of the granulosa cell layer of large antral follicles while some cells of the cumulus cells retain lower AMH expression until the pre-ovulatory stage in the mouse [15]. Initial studies showed that AMH expression in granulosa cells is partially regulated by oocyte-derived signals as in vitro oocytectomy decreases *Amh* mRNA levels [15]. Among various oocyte-derived molecules involved in the follicular maturation, growth differentiation factor 9 (GDF9) and bone morphogenetic factor 15 (BMP15) are found to act in synergy to upregulate AMH synthesis in human [68] and murine granulosa cells [69]. In both species, FSH counteracts the effects of GDF9 and BMP15 on AMH production, preventing AMH overexpression [68, 69]. Chronic treatment with AMH promotes an overall accumulation of small growing follicles through the inhibition of FSH actions and steroidogenesis, which are responsible for the abnormal ovulatory cycles in mice. The lack of AMH production in *Amh*^*−/−*^ female mice leads to a progressive decline of the primordial follicle pool accompanied by a substantial increase of the small atretic and non-atretic follicles during early postpubertal period followed by a robust decrease of the number of these two types of follicles [57]. Although primordial follicles do not express either AMH or AMH receptors, these observations might be the result of an exhaustion of the primordial reserves through a dysregulated recruitment from primordial follicles to growing follicles as *Amh*^*−/−*^ female mice have higher number of small and large pre-antral follicles than wild-type littermates [70]. It is unlikely that this dysregulated recruitment of growing follicles in AMH null mice is due to neuroendocrine defects because these animals have an enhanced and premature recruitment even in the presence of lower FSH levels [70]. Although there is a lack of information regarding the profile of GnRH/LH secretion in *Amh*^*−/−*^ female mice, the evidence that these animals have normal counts of corpora lutea suggests that possible defects upstream in the HPG axis do not justify the progressive decline of the primordial follicle pool. This may be related to an enhanced FSH sensitivity within the gonad as reported by Durlinger and colleagues [71]. Together, these observations indicate that AMH is involved in the control of small follicle pool by inhibiting two steps of folliculogenesis: (1) the initial recruitment from primordial to small pre-antral follicles, and (2) the gonadotropin-mediated recruitment of small antral follicles towards preovulatory stages (Fig. 2c).

AMH may also contribute to the regulation of follicular growth by inhibiting the action of intrafollicular growth factors. Studies employing rat primary ovarian cultures revealed that AMH blocks the stimulatory effects of fibroblast growth factor (FGF), Kit ligand (KITL) and keratinocyte growth factor (KGF) on follicular growth [72]. Further microarray analysis showed that more than 700 transcripts were changed in AMH-treated ovaries. In particular, AMH robustly suppressed genes of the TGF-β and MAPK signaling pathways, and those regulating FGF and KITL signaling [72], which jointly inhibit cell proliferation and follicle transition processes. Although the majority of growth factors were found to be downregulated, vascular endogenous growth factor (VEGF) and growth differentiation factor 1-like (GDF1-like) were upregulated by AMH treatment [72], which requires further investigations to elucidate their role in control of follicular growth.

Functional roles of AMH curbing the gonadotropin-mediated recruitment of small antral follicles may occur through various cellular and molecular mechanisms, which are not fully deciphered. Granulosa cells of small antral follicles initiate a progressive synthesis of estrogens through the enhanced expression of aromatase, which converts androgens into estradiol, through FSH actions in the ovary. To date, it is recognized that AMH dramatically suppresses ovarian aromatase activity even during gestational period, when basal steroidogenic activity is supposed to be low [73]. In addition, in women undergoing assisted reproduction procedure, the intrafollicular concentration of AMH is negatively correlated with estradiol in small antral follicles [74]. Thus, AMH counteracts FSH actions in the ovary by directly regulating the steroidogenic pathway through the regulation of estrogen synthesis. More recently, evidence from studies using prepubertal mice demonstrated that preantral AMH expression and AMH circulating levels increase with peak levels being detected between postnatal day (PND) 17 and 21, which are 2–3 times higher than adult females [60]. Interestingly, during minipuberty, which occurs between PND 12 and 14 in female mice, AMH expression is low and parallels with high FSH actions in the ovary [60]. Treatment with Ganirelix, a GnRH antagonist, during this time window, reverses this expression pattern by increasing *Amh* transcript levels in preantral follicles [60]; however, this may not be due to estradiol-mediated actions in the ovary. Although GnRH signaling blockade decreases intrafollicular estradiol synthesis, administration of physiological doses of estradiol did not alter AMH expression in Ganirelix-treated mice [60], suggesting the FSH actions on the downregulation of AMH may be direct.

To date, these studies depict AMH as a gatekeeper hormone of the ovarian follicular development by favoring pre-antral follicle growth while inhibiting antral follicle maturation. However, it is important to note that AMH priming robustly increases ovulation rate following superovulation stimulation regime in mice [26]. A proposed model by Hayes and colleagues reports that AMH inhibits the following stages of follicular development: recruitment of primordial and antral follicles, atresia and FSH-sensitivity [26]. These stages are commonly controlled by AMH-induced expression of microRNAs, particularly miR-181a and miR-181b, which ultimately downregulate intracellular cascades in granulosa cells [26]. In rodents AMH seems to restrain both pre-antral and early antral stages of the follicular maturation [57, 71], whereas, in primates, AMH inhibitory actions are mostly effective during antral follicle maturation [75]. In addition, non-human primate individual pre-antral follicles respond differently to AMH [75]. These observations suggest the existence of species-specific AMH actions on follicular development and dominant follicle selection.

Recent data from a small clinical study shows that exogenous GnRH lowers circulating AMH levels while increasing FSH and LH levels within 30 min of intravenous administration, which is independent of sex or age [76]. As this effect is fast, it is unlikely that the decrease of AMH levels were due to changes in gonadal steroid hormone actions within the gonad, but they may rather involve direct GnRH actions at the gonadal level.

All these possible mechanisms of AMH actions in the ovary reassure its role as gatekeeper of the ovarian follicle development, and disturbances on ovarian AMH production may contribute to or drive common endocrine disorders affecting reproduction. As described above, AMH acts in the brain and pituitary gland modulating GnRH and gonadotropin release, which ultimately coordinates ovarian function. These direct and indirect gonadotropin-mediated actions indicate that AMH is pivotal for ovarian follicular development and fertility. Future research may define whether the contribution of central and gonadal AMH actions contribute equally to female reproductive competence, or whether there is some degree of critical requirement to achieve normal functioning of the HPG axis.

### AMH integrates androgen and endocrine signals in the testes

The HPG axis orchestrates testicular function over adult life granting the tight regulation of spermatogenesis and androgen production by the testes. Nonetheless, testicular AMH production reaches its maximal levels during embryonic life when sex differentiation occurs. Testicular AMH actions mostly depend upon the developmental stage of Sertoli cells. These cells are the testicular target of FSH actions sustaining spermatogenesis. In mice, androgens inhibit testicular AMH production during perinatal and early postnatal stages, and this progressively declines until PND 15, whereas testosterone secretion into the peripheral circulation increases toward pubertal onset around PND 30 [77]. Intratesticular testosterone content, however, is already increased at birth in mice [77] posing a hurdle in the relationship AMH – testosterone effects in Sertoli cells. Immunohistological data suggested that what impedes the testosterone-mediated inhibition of postnatal AMH production is an androgen insensitivity due to the lack of androgen receptor (AR) expression in Sertoli cells during early stages. AR expression will only increase from PND 7 onward in male mice [77]. This was also known to be the case in men, as studies with biopsies from fetal to adult human testes demonstrated that androgen insensitivity during early stages of development are associated with the lack of AR expression in Sertoli cells, when AMH production is high [78].

Prepubertal FSH-deficient transgenic mice display a lower testicular mass due to a decreased number of Sertoli cells, and both parameters are reinstated to normal levels when animals receive treatment with recombinant FSH for six days [79]. These mice also show lower AMH plasma levels, which are resumed following the administration of FSH [79]. This observation suggests that FSH actions over prepubertal period, including minipuberty, are critical to sustain normal intratesticular AMH levels; thus, coordination of Sertoli cell maturation and spermatogenesis are constantly kept in check.

Sertoli cells secrete AMH into the seminiferous tubules reaching higher concentrations in the seminal fluid when compared with the peripheral blood [80]. There is an inverse relationship between the amount of AMH concentration in the seminal fluid and sperm motility index in humans [80], alluding to a paracrine role of AMH in the maturation and maintenance of germ cells in males. Immature Sertoli cells also express AMHR2 [14] and different AMHR1 receptors, predominantly ALK2 and ALK3 [81], through which AMH activates Smad1 pathway-dependent suppression of gene transcription of *P450scc* and *amhr2* in these cells [81]. The former molecule encodes a key mitochondrial enzyme that converts cholesterol to pregnenolone. It is possible to speculate that the downregulation of steroidogenesis and downregulation of *Amhr2* expression may protect Sertoli cells from overstimulation and from engaging oncogenic processes, similarly to what has been shown in other reproductive tissues [82].

Testosterone is produced by Leydig cells under the modulation of LH actions in the gonad, and these cells express AMHR2 [58]. Transgenic male mice overexpressing human AMH (hAMH) exhibit decreased testosterone levels, although LH levels are substantially high in these animals [58]. Thus, high intragonadal AMH levels may impair LH-mediated androgen production in the testes of these animals. Morphometric analysis of the testicular tissue of hAMH mice revealed a lower number of immature and mature Leydig cells whereas an increased number of precursor mesenchymal cells [58], suggesting that AMH may gatekeep the maturation of Leydig cells from its mesenchymal source over fetal development. AMH also interferes with LH in the control of androgen production directly in Leydig cells. For instance, a single injection of AMH in adult male mice decreases the levels of LH receptor (LH-R) expression [83] and of the enzymes that control the biosynthesis of androgens in AMH-treated purified Leydig cells [58, 83]. These findings corroborate the idea that AMH stalls both precursor mesenchymal cells differentiation into Leydig cells and steroidogenesis regulating testicular androgen production (Fig. 2d).

## AMH and reproductive pathophysiology

AMH is a key hormone attaining reproductive function in both sexes in normal conditions. As expected, genetic or environmentally induced alterations in AMH signaling may trigger diseases affecting sexual development and fertility. In this session, our review will highlight current views about the putative participation of AMH in either the etiology or maintenance of different neuroendocrine reproductive disorders.

### Polycystic ovary syndrome (PCOS)

PCOS is the main endocrine disorder leading to female infertility worldwide, affecting around 5 to 20% of women in the reproductive age [84, 85]. The disease is characterized by three cardinal features according to the Rotterdam, Androgen Excess and PCOS (AE-PCOS) Society, and National Institutes of Health (NIH) criteria: androgen excess, menstrual irregularities and polycystic-like ovarian morphology (PCOM). According to the Rotterdam criteria and clinical consensus, upon the presence of two of those three features, PCOS may be diagnosed [86]. Current view indicates that PCOS may be primarily rooted in the effects of androgen excess and its consequences to fertility [87, 88]. PCOS is also commonly associated with other comorbidities such as metabolic syndrome [89, 90], depression and anxiety [91], and cancers of the reproductive tract [92]. This heterogeneity is suggestive of different etiological backgrounds yet the mechanisms underlying its pathogenesis remain unclear.

Although PCOS has been classically considered an ovarian disorder [93, 94]; clinical and pre-clinical studies strongly endorse that neuroendocrine derangements may play a role in its etiology [95]. Early discoveries presented that PCOS patients exhibit high LH levels in the blood [96, 97], and enhanced response to GnRH administration compared with healthy subjects [98]. High LH levels are the reflex of an exaggerated pulsatile LH release without affecting FSH secretion in hyperandrogenic PCOS women [99, 100]. Although these characteristics indicated that impaired gonadotropin secretion occurs in PCOS, there was no clear evidence whether those abnormalities were caused by defects in the brain or in the pituitary gland. Daniels and Berga observed that, while healthy women under standard oral contraceptives regime display suppressed LH pulse secretion, PCOS patients still show LH pulses [101]. They also reported that LH pulse frequency increased even higher in these patients following the withdraw of the contraceptive regime [101]. These observations indicate that there is a resistance of the GnRH pulse generation to steroid hormone-mediated negative feedback leading to an intrinsic enhanced GnRH drive in the PCOS brain.

A more recent development in this search for clarification of the etiology of PCOS is the involvement of AMH. Serum AMH levels are frequently elevated in PCOS women, around 2 to fourfold higher, when compared with normal ovulatory women [102–105]. In addition, these high AMH levels persist and increase throughout pregnancy in women with PCOS compared with weight-matched non-PCOS controls [43, 106]. Elevated circulating AMH levels in women with PCOS are mainly due to large numbers of secondary preantral and small antral follicles. Within PCOS, circulating AMH levels reflect the severity of PCOS phenotype, being higher in anovulatory than ovulatory PCOS patients [107, 108].

Elevated plasma AMH levels are surrogate for menstrual irregularities [104] and they are robustly associated with high LH levels in PCOS patients [109, 110]. Interestingly, in naturally occurring hyperandrogenic non-human primate model of PCOS, both AMH and LH levels are elevated [111]. The source of augmented AMH production in PCOS is likely to be the granulosa cells of PCOS ovaries. As detailed in previous sections of this review, AMH actions in granulosa cells decreases the FSH- and LH-induced expression of aromatase (cytochrome P450) decreasing estradiol production while favoring androgen accumulation [112]. In addition, small antral follicle granulosa cells from anovulatory PCOS patients present a premature response to gonadotropins when compared to healthy controls [113], which may contribute to the loss of homeostatic control of gonadotropin-mediated ovarian steroid production in PCOS. Exposure of human ovarian granulosa‑like cell line KGN-GC, resembling granulosa cells from smaller antral follicles, to hyperandrogenemic levels of dihydrotestosterone (DHT) prevents the expected decrease of AMH production [114]. Together with the accumulation of small pre-antral follicles providing the high increment in AMH secretion [107], this abnormal desensitization of granulosa cells, hyperandrogenemic milieu and enhanced LH actions in the ovaries may be culprit for elevated AMH and actions in PCOS.

In recent years, researchers started to investigate whether abnormal levels of circulating AMH might affect the neuroendocrine circuits regulating reproduction. As described in previous sections of this review, AMH robustly increases GnRH neuron activity, and intracerebroventricular administration of AMH in mice leads to enhanced LH pulse frequency release [6]. Using a prenatally androgenized (PNA) mouse model of PCOS, researchers demonstrated that while it remains unclear whether circulating AMH levels are increased in these animals, AMH and LH levels are positively correlated, which is not the case in control mice [6]. PNA mice are hyperandrogenic, present high LH pulse frequency and intrinsic defects in the estradiol- and progesterone-mediated negative feedback [115, 116], such as in the human condition [117].

Prenatal androgen exposure has been considered a strong driver of the manifestation of PCOS features following pubertal onset. Conditions such as congenital adrenal hyperplasia (CAH) [118] and PCOS itself [119, 120] are known to promote higher androgen levels during pregnancy, and both conditions increase the chance of daughters to present PCOS features during adulthood. In order to mimic the human pathology, researchers have mostly used animal models including nonhuman primates [121], sheep [122] and rodents [123] to study the influence of the prenatal androgen exposure in the onset of PCOS. Although this extensive and rising number of reports describing the effect of prenatal androgen exposure affecting the female brain will not be discussed in detail in this review, it is important to note that prenatal exposure to androgen actions is probably one of the main causes driving PCOS adult manifestation [124, 125].

**Fig. 3:**
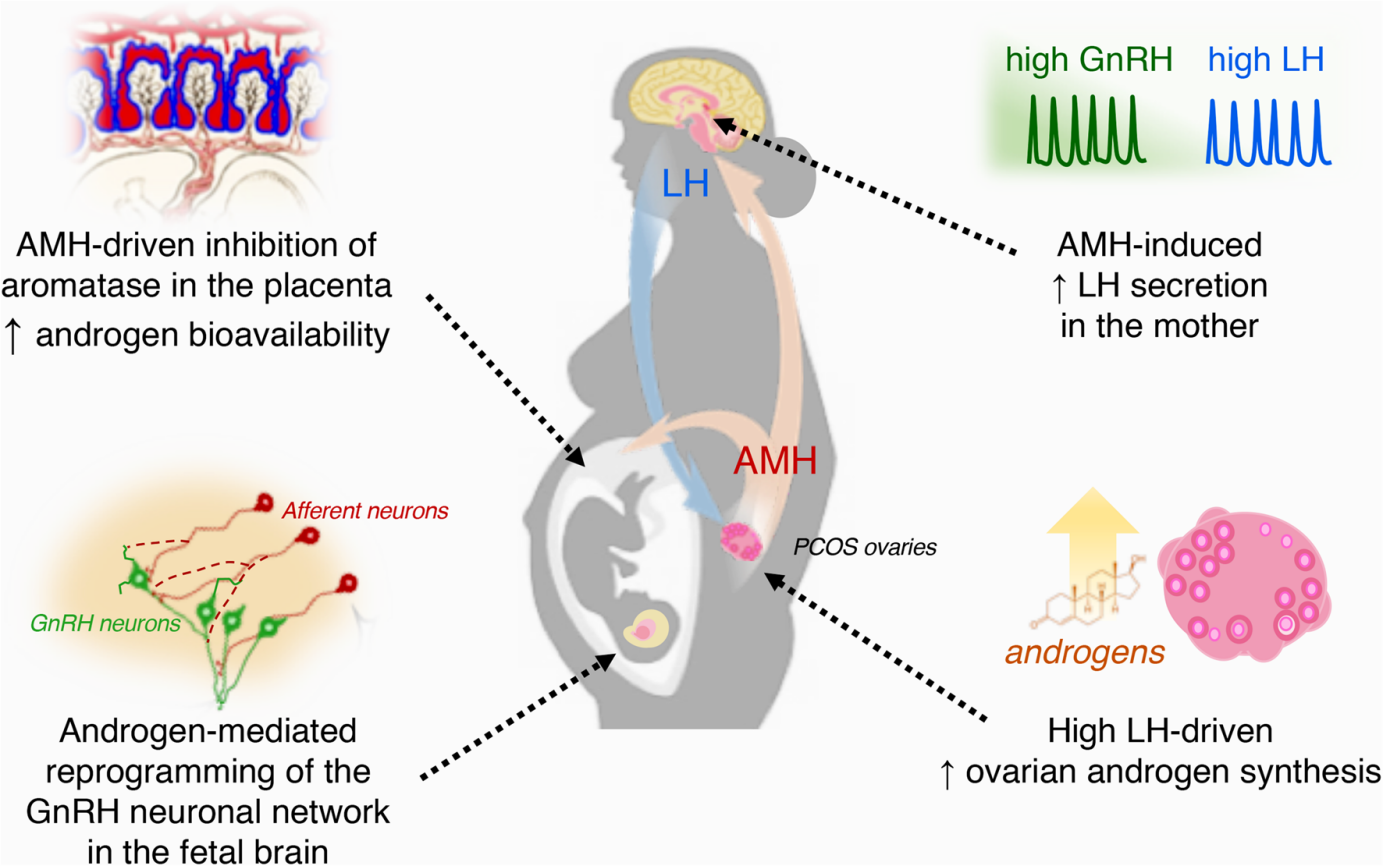
AMH prenatal programing in the pathophysiology of PCOS. Clinical evidence indicates that PCOS mothers maintain high AMH levels from the second gestational trimester to term. Maternal AMH-induced changes in PCOS may rely on central actions that increase GnRH pulsatile release, hence, culminating in high LH pulsatile secretion. Enhanced LH actions in the PCOS ovaries favor ovarian androgen production creating a hyperandrogenic environment. AMH also inhibits maternal placental aromatase expression, which increases testosterone bioavailability in utero. Prenatal and neonatal hyperandrogenism leads to increased excitatory GABAergic appositions to GnRH neurons and to a persistent GnRH neuronal hyperactivity in adulthood. Excitatory innervation of GnRH neurons might be responsible for the increased GnRH–LH pulse frequency observed in PCOS-like animals. These abnormal changes may dictate the neuroendocrine derangements of the adult manifestation of PCOS and may play an important role in the etiology of the disease

A recently noted action at the hypothalamic level shows that AMH may enhance GnRH release in normal and PCOS-like conditions [6]. AMH receptors are expressed in human GnRH neurons and AMH can directly increase GnRH-dependent LH secretion [6]. These findings imply that high ovarian AMH levels in women with PCOS can regulate both ovarian follicle development and hypothalamic GnRH release. More recently, it has been shown that prenatal exposure to high AMH over late gestation in mice drives gestational hyperandrogenism via inhibition of placental aromatase [43]. These dams give birth to female offspring (PAMH mice) that recapitulate all cardinal features of PCOS, reduced fertility and neuroendocrine derangements such as high GnRH neuronal activity and high LH pulsatile secretion [43]. Researchers also found that PAMH treatment promotes the remodeling of placental steroidogenesis resulting in the inhibition of aromatase expression [43], hence increasing the bioavailability of testosterone in utero. AMH also accesses the pregnant female mouse brain through the median eminence, a blood–brain barrier (BBB)-free hypothalamic site [43]. AMH-induced androgenization may also rely on central actions of AMH enhancing GnRH release as PAMH dams also present high LH and testosterone levels at term [43], such as in the human condition [106] (Fig. 3).

From midgestation to term, embryonic circuits that later will orchestrate reproductive function are being shaped in the female brain. In fact, organizational effects driven by gonadal steroid hormones (e.g. estradiol and testosterone) promote the molecular programing of reproductive circuits that will influence the activation of the HPG axis sexual behavior following pubertal development [126]. Adult PAMH female mice present high GnRH neuron activity and an enhanced GABAergic tone onto GnRH neurons [43], which have been associated with high GnRH/LH secretion and the presence of PCOS-like features in mice [46, 116, 127]. PAMH mice also display virilization of kisspeptin and dopaminergic neurons in the anteroventral periventricular hypothalamus (AVPV), which are important for the control of GnRH neuron activity and LH-surge mediated ovulation, vasopressin neurons of the bed nucleus of the lamina terminalis (BNST), which are engaged in sexual behavior [43]. These discoveries suggest that AMH-dependent gestational hyperandrogenism reprograms hypothalamic circuits culminating in impaired reproductive fitness in the PCOS progeny (Fig. 3).

Maternal inheritance of PCOS has fivefold increased risk when compared with daughters born from health control mothers [128]. AMH levels are already significantly higher in PCOS daughters from early infancy (2–3 months old) [129] to prepubertal and peripubertal periods [103, 129, 130]. Interestingly, recent studies have identified gene variants of *AMH* and *AMHR2* being associated with ~ 3% of familial PCOS cases [131, 132]. These findings suggest that AMH signaling is involved in the pathogenesis of PCOS, even though more studies investigating the contribution of rare genetic variants in PCOS are required. The regulation of gene expression is also achieved through epigenetic modifications, which may contribute to the transgenerational susceptibility of PCOS. In fact, a recent clinical study using targeted next-generation sequencing (NGS) reports that sons born to PCOS women present lower methylation of CpG sites in three regions of the AMH promoter compared with sons of non-PCOS mothers [133]. Hypomethylation marks are usually an indication of higher promoter activity; therefore, this evidence may account to the high levels of AMH in PCOS sons throughout childhood [134]. Although these epigenetic marks were not detected in PCOS daughters, researchers discovered that these girls present hypomethylation of two CpG sites in two regions of the androgen receptor (AR) promoter [133]. Thus, increased plasma levels of AMH in PCOS daughters could be secondary to alterations in androgen signaling and altered follicular development after puberty.

To conclude, since neuronal AMH and AMHR2 expression have been associated with embryonic neuronal differentiation in both humans and mice [6, 11] and that PAMH female mice exhibit many PCOS-like traits [43], animal models expressing altered AMH or AMHR2 expression, including genetically manipulated and PAMH mice, together with naturally occurring PCOS-like non-human primates, potentially promise valuable mechanistic insights into this disease.

### Congenital hypogonadotropic hypogonadism (CHH)

CHH is a rare genetic disease of sexual maturation caused by the chronic deficiency in the production or secretion of GnRH. Although the prevalence of CHH remains uncertain, it is believed to affect around 1/5000 men, which can be three to five times less frequent in women [135]. In CHH patients, the HPG axis is not activated leading to hypogonadotropic hypogonadism, lack of puberty onset and infertility [136, 137].

Around 50–60% of CHH individuals present anosmia, loss of sense of smell, and this condition is termed Kallmann’s Syndrome (KS), whereas CHH patients with normal olfaction are diagnosed as normosmic congenital hypogonadotropic hypogonadism (nCHH) [136]. GnRH deficiency and anosmia co-occur in KS as GnRH neurons do not migrate properly into the forebrain due to olfactory bulb aplasia, abnormal palate morphogenesis and intrinsic defects in the GnRH neuron system during migration [138, 139]. CHH is caused either by mutations affecting the migration of GnRH neurons and leading to a central GnRH deficiency, with GnRH neurons being stuck in the nasal regions, or by mutations affecting GnRH function and signaling pathway [136]. Genetic studies have revealed that KS is genetically heterogenous, which explains the broad range of phenotypes leading to GnRH-deficiency and reproductive impairment [136, 139]. Novel evidence by Malone and colleagues demonstrated that both AMH and AMHR2 are expressed during embryonic development by migratory GnRH neurons and that AMH acts as a pro-motility factor on GnRH cells and that acute neutralization of AMHR2 or genetic invalidation of this receptor in mice leads to a migratory defect of GnRH neurons and defects in the intracranial projections of the VNN/TN [11]. These alterations resemble the phenotype previously described in pathohistological analyses of KS human fetuses [140]. The same authors have then performed whole-exome sequencing in a large cohort of CHH probands focusing on AMH and its exclusive binding receptor, AMHR2, identifying several missense mutations, all in the heterozygous state [11]. The low penetrance of most CHH associated genes combined with variable phenotypic presentation among affected individuals carrying the same genetic defects suggests that CHH is not strictly a monogenic disorder [141, 142].

In vitro studies in which COS-7 cells were transfected with either KS- or nCHH-derived AMH variants revealed that AMH secretion is substantially decreased [11]. Cell motility assays further revealed that immortalized GnRH neurons, expressing these AMH variants, display decreased motility in response to AMH [11], indicating that these mutations may account for the defective GnRH neuron migration in CHH. Using a secretory cell model of GnRH neurons, GT1-7 cells, researchers discovered that KS- and nCHH-derived AMH mutations also impair GnRH release [11]. The consequences of AMHR2 loss-of-function were also tested revealing that AMHR2 mutations decrease cell migration in GN11 cells and decrease GnRH secretion in GT1-7 cells [11]. These data strongly indicate that AMH and AMHR2 mutations identified in CHH have a pathogenic effect.

In summary, these recent findings provide insight into the molecular basis of AMH-dependent signaling in the correct establishment of the GnRH migratory process and suggest that AMH signaling insufficiency may contribute to the pathogenesis of CHH.

### Persistent Müllerian duct syndrome (PMDS)

The regression of Müllerian ducts is an irreversible phenomenon occurring during a critical time window when the Müllerian ducts are hormone sensitive [143, 144]. Action of AMH on Müllerian ducts’ regression seems to be paracrine, as some case of unilateral regression of the Müllerian ducts have been documented from different mammalian species [145, 146]. The role of AMH and AMHR2 in the appropriate development of genital tract has been confirmed in patients affected by a genetic condition known as persistent Müllerian duct syndrome (PMDS). PMDS is a rare disorder of sexual development, characterized by the persistence of Müllerian ducts derivatives including uterus, fallopian tubes and the upper two-third of vagina, in otherwise normal genotypical (46, XY) and phenotypical (normal virilization of external genitals) men. PMDS men usually suffer from infertility due to two mechanisms which may co-exist: abnormalities in the testicular descent (resulting in cryptorchidism or testicular ectopia) and abnormalities of male excretory ducts. Frequent aplasia of epididymis precluding connection of the *vas deferens* (which is also often abnormal) with the normal differentiated testis was observed in PMDS [147]. Genetic analysis has revealed that this syndrome is due to autosomal recessive inheritance mutations in *AMH* or *AMHR2* genes, with no significant phenotypic difference between men with *AMH* or *AMHR2* mutations [147–149]. During prepubertal life, AMH levels are significantly low in PMDS boys [149]. However, whether AMH deficiency may have also an impact on the HPG axis maturation has not been investigated so far in these patients. Indeed, to date, the lack of both clinical reports studying the neuroendocrine features and animal models resembling the disease have prevented to fill this gap of knowledge.

## Conclusion and perspectives

The emerging roles of AMH in neuroendocrine function and reproduction have uncovered its versatile actions in different levels of the HPG axis. AMH is phylogenetically ancient and classically recognized for its effects on secondary sex determination and gonadal development in males. Rising evidence gives support that while AMH defines the fate of gonadal development in males during embryonic life, this hormone is critical for prepubertal, pubertal and adult reproductive life of females through the control of ovarian follicular development. The central effects of AMH define a new time for research in neuroendocrinology of reproduction. AMH robustly increases GnRH neuron activity in both sexes and effectively drives GnRH secretion from neuronal terminals. As AMH enhances LH pulse frequency release, direct actions in GnRH neurons and indirect modulation of other neurons in the GnRH neuronal network should be further investigated in the future. AMH also facilitates gonadotropin secretion enhancing the gonadotropin sensing of differential GnRH pulsatile release. Central and pituitary regulation may be dependent upon gonadal AMH secretion; however, it becomes clearer that tissular expression of AMH and AMHR2 in neurons and gonadotropes may contribute to the fine tuning of the signaling outcome. Circulating levels of AMH and proAMH may also define how different tissues respond to variations in their concentration and whether extra-gonadal cleavage of proAMH could define tissue-specific modulation of AMH signaling. In this context, new reliable assays to measure different AMH isoforms are needed to get insights into the expression and role of these isoforms in the context of several reproductive disorders. AMH has been implicated in the pathogenesis of PCOS, a highly prevalent disorder leading to female infertility; however, better diagnostic tools and the relationship between AMH levels and PCOS clinical features still demand further improvement [150].
